# The Characters of Dry Soil Layer on the Loess Plateau in China and Their Influencing Factors

**DOI:** 10.1371/journal.pone.0134902

**Published:** 2015-08-04

**Authors:** Weiming Yan, Lei Deng, Yangquanwei Zhong, Zhouping Shangguan

**Affiliations:** State Key Laboratory of Soil Erosion and Dryland Farming on the Loess Plateau, Northwest A&F University, Yangling, Shaanxi, 712100, P.R. China; Chinese Academy of Forestry, CHINA

## Abstract

A dry soil layer (DSL) is a common soil desiccation phenomenon that generally forms at a particular depth in the soil profile because of climatic factors and poor land management, and this phenomenon can influence the water cycle and has been observed on the Loess Plateau of China and other similar regions around the world. Therefore, an investigation of the DSL formation depth (DSLFD), thickness (DSLT) and mean water content (MWDSL) on the Loess Plateau can provide valuable information. This paper synthesized 69 recent publications (1,149 observations of DSLs from 73 sites) that focused on DSLs in this region, and the results indicated that DSLs are significantly affected by climatic and vegetation factors. The mean annual precipitation had a significant positive relationship with DSLFD (p = 0.0003) and MWDSL (p<0.0001) and a negative relationship with DSLT (p = 0.0071). Crops had the lowest DSLT and highest MWDSL values compared with other vegetation types. A significant correlation was observed between the occurrence of DSLs and the years since planting for grasses, shrubs, trees and orchards, and the severity of DSLs increased with increasing planting years and wheat yield. Our results suggest that optimizing land-use management can mitigate DSL formation and development on the Loess Plateau. Understanding the dominant factors affecting DSLs will provide information for use in guidelines for the sustainable development of economies and restoration of natural environments experiencing water deficiencies.

## Introduction

Rainfall patterns are expected to change around the world because of climate change [1], and for a number of regions, including arid and semiarid areas, droughts will become more frequent and severe [2–4]. Global warming is also expected to increase evapotranspiration, which may offset modest increases in precipitation and result in decreased soil moisture content and increased aridity in water-limited systems worldwide [5]. This climate change will result in soil desiccation and may cause the formation of a dried soil layer (DSL) in soil profiles, which is influenced by the distribution of plant roots [6–10] and eventually causes decreased soil water storage, which is an important water source for plants that is used to maintain and regulate growth under drought stress, especially in arid regions that have insufficient rainfall to meet normal growth requirements [10].

The formation of a DSL is a common hydrological phenomenon and primarily results from the high rate of evapotranspiration associated with plants and evaporation from soil combined with long-term insufficient rainfall [6, 7, 11, 12]. A DSL can be described as a soil profile with an extreme deficiency of soil water that may reach wilting moisture levels because of the excessive consumption of water by the plants, and can’t restored even after the rainy season. In general, DSLs have been described as having the following characteristics: (1) layers that exhibit a range of soil water content (SWC) from permanent wilting point to the stable water levels or levels at which water is dispersed by soil capillary action (SFC), which is at approximately 60% of the soil field capacity (FC) based on the texture of soil found on the Loess Plateau [6, 10, 13, 14]; and (2) layers that are located at a certain soil depth and persist for a long time. DSLs have been found in water-deficient regions around the world, such as in eastern Amazonia [11], southern Australia [15] and the Loess Plateau of China [10]. In the dry eastern end of the Amazon basin, DSLs have developed because of the excessive depletion of deep soil water [11]. In South Australia, Richards et al. [16] found that eucalypts have a significant drying effect, whereas pines have little effect. In addition, Li [10] observed that DSLs are a serious problem on the central Loess Plateau of China. However, this finding has not prevented the conversion of farmland to artificial forest or grassland, which has created serious negative effects on soil hydrological conditions because of the long-term water deficiencies associated with DSLs [7, 14, 17]. When a DSL occurs in deeper soil layers, it will greatly reduce the adaptive capability of the “soil reservoir” to supply water from the deep soil layers to plants for continued growth [10, 18–20], thus causing the degradation and death of non-native vegetation, preventing the replanting of vegetation because of deficits in the deeper layer water [21, 22], and influencing the recharge of soil water [15]. DSLs were first observed in the 1960s in farmlands, artificial grasslands and forests in the semi-arid regions on the Loess Plateau of China [10]; however, DSLs have rarely been observed in natural vegetation. Replacing farmland with forest or grass has led to a considerable increase in vegetation coverage, but large-scale vegetation restoration has also aggravated soil water scarcity and promoted a low survival rate of trees and “small aged trees” [23, 24], and it has also led to the formation of DSLs in the deep soil layers [13, 19]. Therefore, in recent years, studies on DSLs have emphasized assessments of their basic characteristics [6, 17, 19, 25, 26], types [6, 10, 17], and formation mechanisms [17, 27, 28], number of studies have focused on the presence of DSLs on the Loess Plateau because they are more serious and widespread than in other regions due to the unique climate, low water tables and poor land management [12].

Although the natural conditions of climate, hydrology and soil may be responsible for the common nature of DSLs, non-native vegetation restoration still has significant effects on DSLs [6, 7, 17, 29]. DSLs are more serious in areas covered by trees and shrubs than in areas covered by grasses because of the high evapotranspiration rates associated with tree and shrubs, and they are more serious in the land covered by high-productivity than in the land covered by low-productivity land [6, 19]. In addition, DSLs are associated with vegetation age, with the deepest DSLs in *Medicago sativa* L. grassland in Jiatang County reaching 540, 580 and 900 cm after 3, 4 and 6 years of growth, respectively [30]. Cheng et al. [31] found that DSLs did not occur within 0–800 cm in soil associated with the young growth stage of *Caragana korshiskii* Kom, whereas in the middle and older growth stages, the DSL thickness (DSLT) reached 260 and 700 cm, the DSL formation depth (DSLFD) was 220 and 80 cm, and the mean water content of the DSL (MWDSL) was 7.17% and 6.48%, respectively. Moreover, the deepest DSL layer in alfalfa grasslands reached 2,000 cm within 23 years after planting [32]. Previous research has reported that the duration of SWC recovery varied from 6.5 to 19.5 years (average 13.7 years) in the 0–1,000 cm soil layer and from 4.4 to 8.4 years (average 7.3 years) in the upper 0–300 cm soil layer after a 30-year apple orchard was converted to winter wheat on the Loess Plateau of China due to the unique climate and physiognomy [33].

Currently, a number of studies have focused on the DSLs (including DSLT, DSLFD and MWDSL) on the Loess Plateau of China, but their scopes are rarely on a large scale [7, 12, 18]. Thus, investigations of the severity of DSLs under different climate conditions (primarily precipitation variations) within a large-scale area and the influence of vegetation types and their growth ages is necessary. Therefore, the threefold objectives of this study were to (1) measure the DSLT, DSLFD and MWDSL under different vegetation types and relationship with precipitation; (2) explore the relationship between DSLT, DSLFD and MWDSL and the planting years of different vegetation types; and (3) analyse the DSLs status of orchard and alfalfa and relationship with wheat yield.

## Methods

### Description of the study area

The Loess Plateau of China (spanning 100°54′–114°33′E and 33°43′–41°16′N) covers a total area of 62.85×10^4^ km^2^ at an elevation of 1200–1600 m above sea level. The region is covered with thick loess deposits largely ranging from 30 to 80 m in thickness [34]. The Loess Plateau is located in a transitional zone that extends from southeastern humid monsoon climates to northwestern continental dry climates, and it is famous for its deep loess deposits, unique landscapes, and intense soil erosion (1000–15,000 t km^−2^ a^−1^) due to low vegetation coverage [35]. The intense soil erosion has instigated a series of environmental problems, including environmental degradation, decreasing land productivity, and downstream riverbed uplift of the Yellow River [35, 36]. Therefore, numerous afforestation campaigns, costing hundreds of billions of Yuan have been implemented to control the soil erosion [12, 24].

### Data preparation and collection

The relevant literature (2000–2014) on DSLs on the Loess Plateau was searched using the online databases of the Chinese Academy of Sciences (http://www.isiknowledge.com/ and). A Preferred Reporting Items for Systematic Reviews and Meta-Analyses (PRISMA) checklist was applied (S1 Table). In total, the final dataset contained 69 studies, including 1,149 observations of DSLFD, DSLT and MWDSL that fit our selection criteria from 73 sites, with the units of DSLT and DSLFD expressed in cm and units of MWDSL expressed in g g^-1^. The raw data were obtained from tables or extracted by digitizing graphs using the GetData Graph Digitizer (version 2.24, Russian Federation) when the data were only expressed in the form of figures. The following information was compiled: data source(s), location (longitude and latitude), climatic information (mean annual temperature and precipitation from the literature or weather stations), vegetation type (grass, shrubs, trees, orchards and crops), and planting years. To avoid distortions caused by publication dates, the selected data were held to the following criteria: (i) the study sites must be located on the Loess Plateau and feature no irrigation; (ii) the depth of the soil must not be less than 600 cm; and (iii) the studies must have been published between 2000–2014.

**Fig 1:**
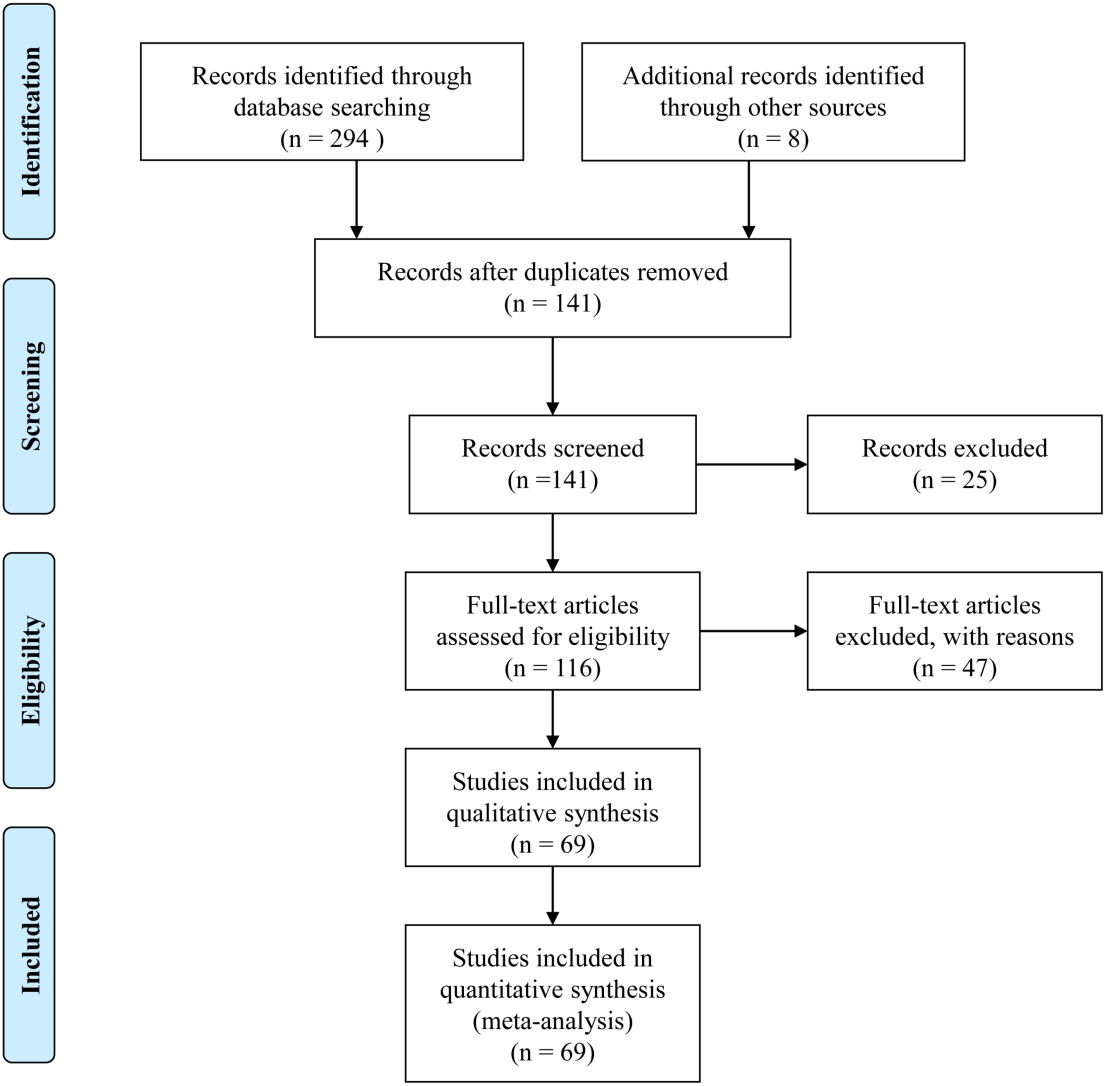
Flow diagram reporting the number of records identified, excluded, and added during the screening process.

The literature search was performed in October 2014, and a total of 294 studies were retrieved, although additional studies were obtained from colleagues. After removing duplicates, 141 studies remained. The studies were selected in a two-stage process. First, 116 studies with relevant titles were selected. Second, selection was made based on abstracts and full paper content. After selection, 69 studies remained (Fig 1, see S1 File for database and references).

**Fig 2:**
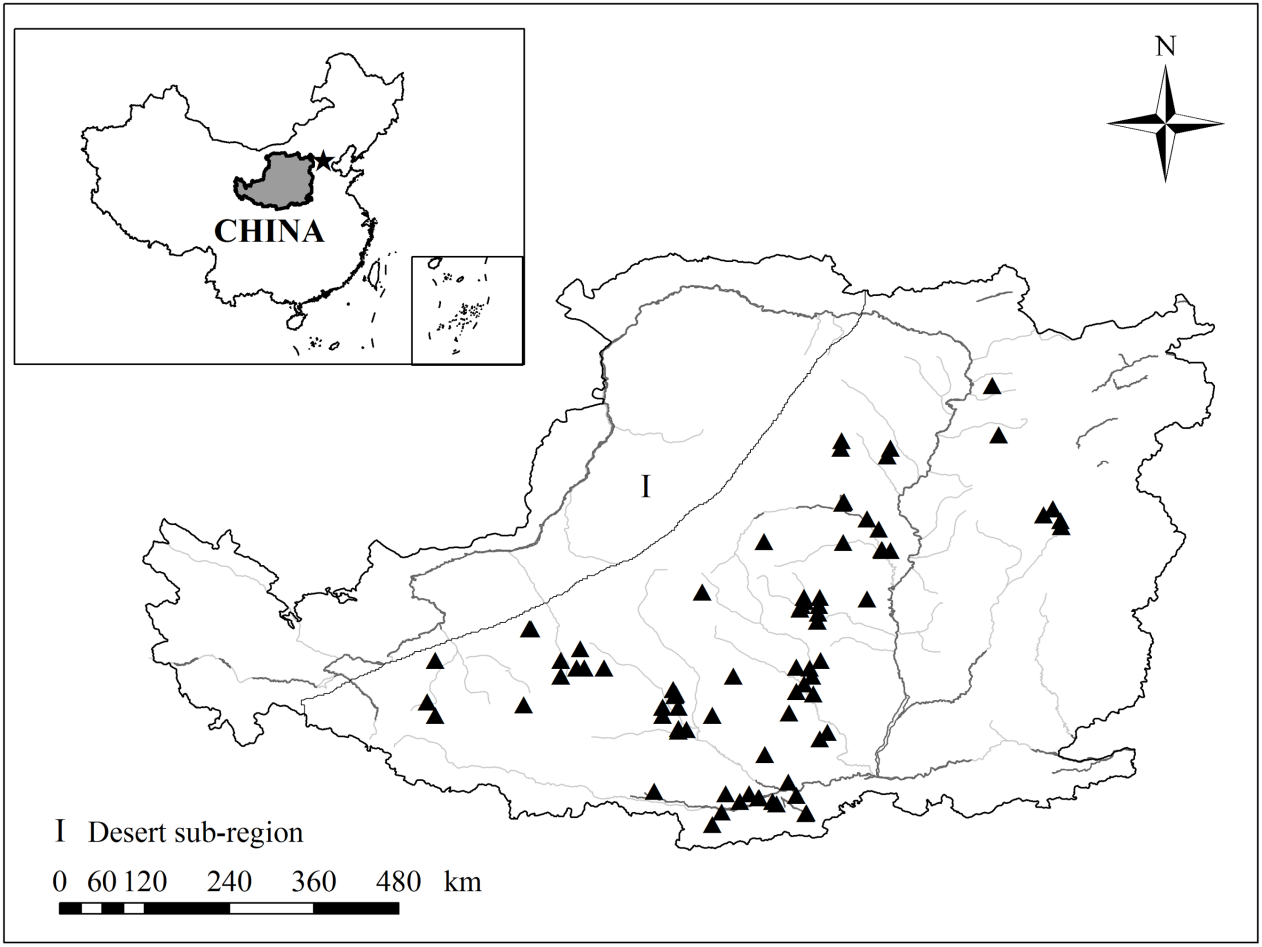
Location of the Loess Plateau in China and the distribution of sampling sites on the Loess Plateau.

The collected data for the DSLs, including data on DSLT, DSLFD and MWDSL, for sampling sites in the provinces Shaanxi, Gansu, Ningxia, and Shanxi on the Loess Plateau are shown in Fig 2, which was created using ArcGIS 9.3 software. In this study, the vegetation was divided into five types according to plant characteristics: grass, shrub, tree, orchard and crop.

### Statistical analysis

A one-way analysis of variance (ANOVA) was performed to determine the significance of the differences observed among the five vegetation types. Significant differences were evaluated at the 95% confidence level. All of the statistical analyses were performed using the software program SPSS, ver. 20.0 (SPSS Inc., Chicago, IL, USA).

## Results and Discussion

### Relationship between DSL characters and plant types and precipitation

**Fig 3:**
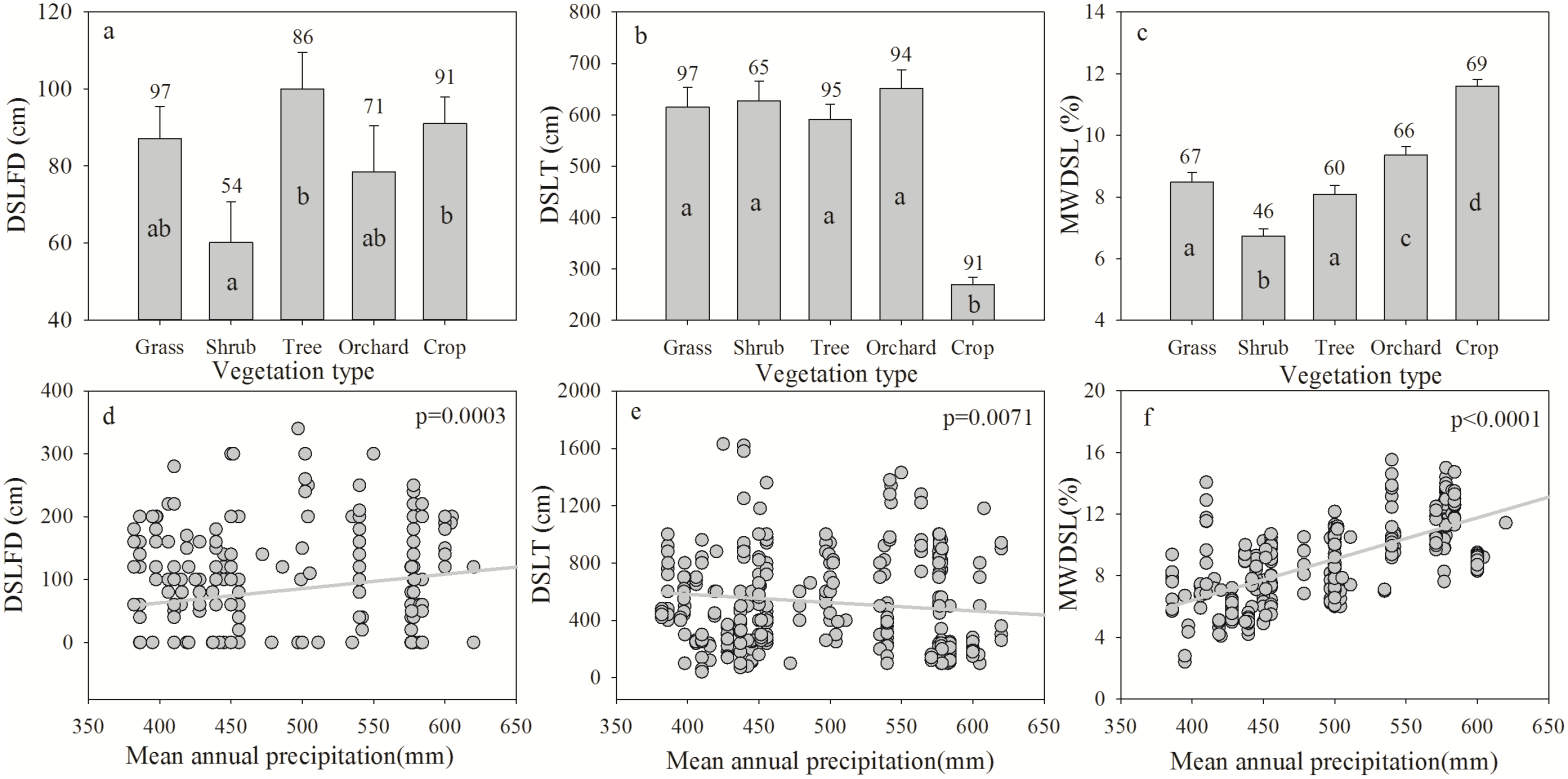
Dry soil layer formation depth (DSLFD), dry soil layer thickness (DSLT) and mean water content of the dry soil layer (MWDSL) for vegetation types and relationships with mean annual precipitation. The lowercase letters within the vertical bars indicate significant differences at α = 0.05 according to the LSD test.

The impact of land use on DSLs differs among the five vegetation types, with shrubs showing the lowest DSLFD values (60 cm), crops featuring the lowest DSLT values (270 cm), and crops and shrubs presenting the highest MWDSL values (11.60%) and lowest MWDSL values (6.73%), respectively (Fig 3a–3c). Crops experienced less DSLs because crops have shallower roots, lower transpiration rates and shorter growing periods, which lead to less water consumption relative to the other vegetation types [37]. The reported degree of DSLs under natural vegetation is generally less severe than that under non-indigenous plant species, and vegetation allowed to follow natural succession process produces fewer DSLs during vegetative growth, which is primarily because natural vegetation maintains a water balance according to the changing local climate in this region [7, 38, 39]. To control soil erosion, farmland has been replaced with forest or grass in large areas of the Loess Plateau. However, the annual precipitation is less than 650 mm, and trees or other vegetation types with high rates of water consumption (such as *Robinia pseudoacacia* L., *Medicago sativa* L., *Astragalus adsurgens* Pall.) have been planted for economic profits, which has accelerated the development of DSLs in this region [6, 17]. Wang et al. [37] reported that the SWC values of farmland and natural grassland are higher than non-native vegetation and that the SWC associated with artificial shrubs (*Caragana korshiskii* Kom) is lower than that of artificial trees (*Platycladus orientalis* L. and *Pinus tabulaeformis* Carr.) in Suide County, Shannxi Province. To a large extent, the rate of soil water loss by plant transpiration depends on climatic conditions and vegetation types. Different plants have different root distribution patterns, water uptake abilities and stomatal conductances [40], which may cause different patterns of DSL development among different vegetation types. Climate change and poor land management, including the introduction of non-native plant species, has accelerated the severity of DSL development on the Loess Plateau [37, 39, 41].

The results show that the mean annual precipitation has a significant positive relationship with DSLFD (p = 0.0003) and MWDSL (p<0.0001) and a negative relationship with DSLT (p = 0.0071) (Fig 3d–3f). These findings indicate that DSL development is more severe in low-precipitation regions of the Loess Plateau because precipitation is the only source of soil water input, are consistent with the results of previous studies [8, 17, 39]. Studies have reported that DSL development becomes more intense and is exhibited across a greater range in depth with lower water content, which has a close negative relationship with precipitation [17, 19, 42]. In high-precipitation regions, the excess amount of precipitation is stored in the soil as a water source for plant growth when precipitation is insufficient. However, on the Loess Plateau, where features intense plant transpiration and soil evaporation, precipitation cannot meet the water requirements of the plants. Thus, vegetation absorbs water from the “soil water pool,” which is located at depths that are not reached by precipitation. Therefore, thicker DSLs and lower MWDSL values are observed in low-precipitation regions. The climate warming trend from 1961 to 2000 coincided with a decrease in the mean annual precipitation by 2.095 mm per year on the Loess Plateau; however, higher temperatures also increase water loss through evapotranspiration, and both climactic changes will lead to the development of more serious DSLs in this region in the future [41].

### Dynamic variation of DSLs with vegetation planting time

**Fig 4:**
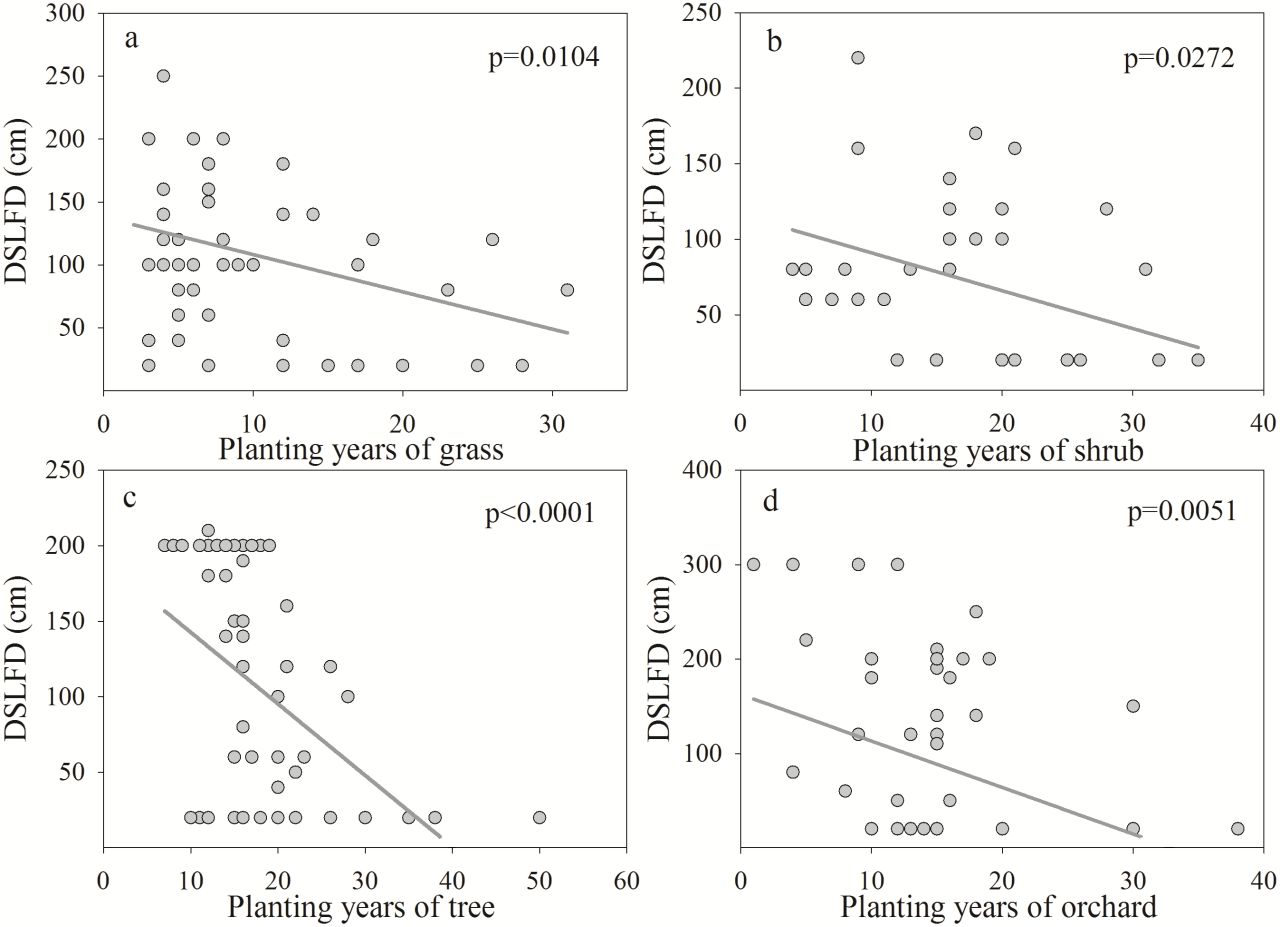
Relationship between the dry soil layer formation depth (DSLFD) and planting years for different vegetation types.

**Fig 5:**
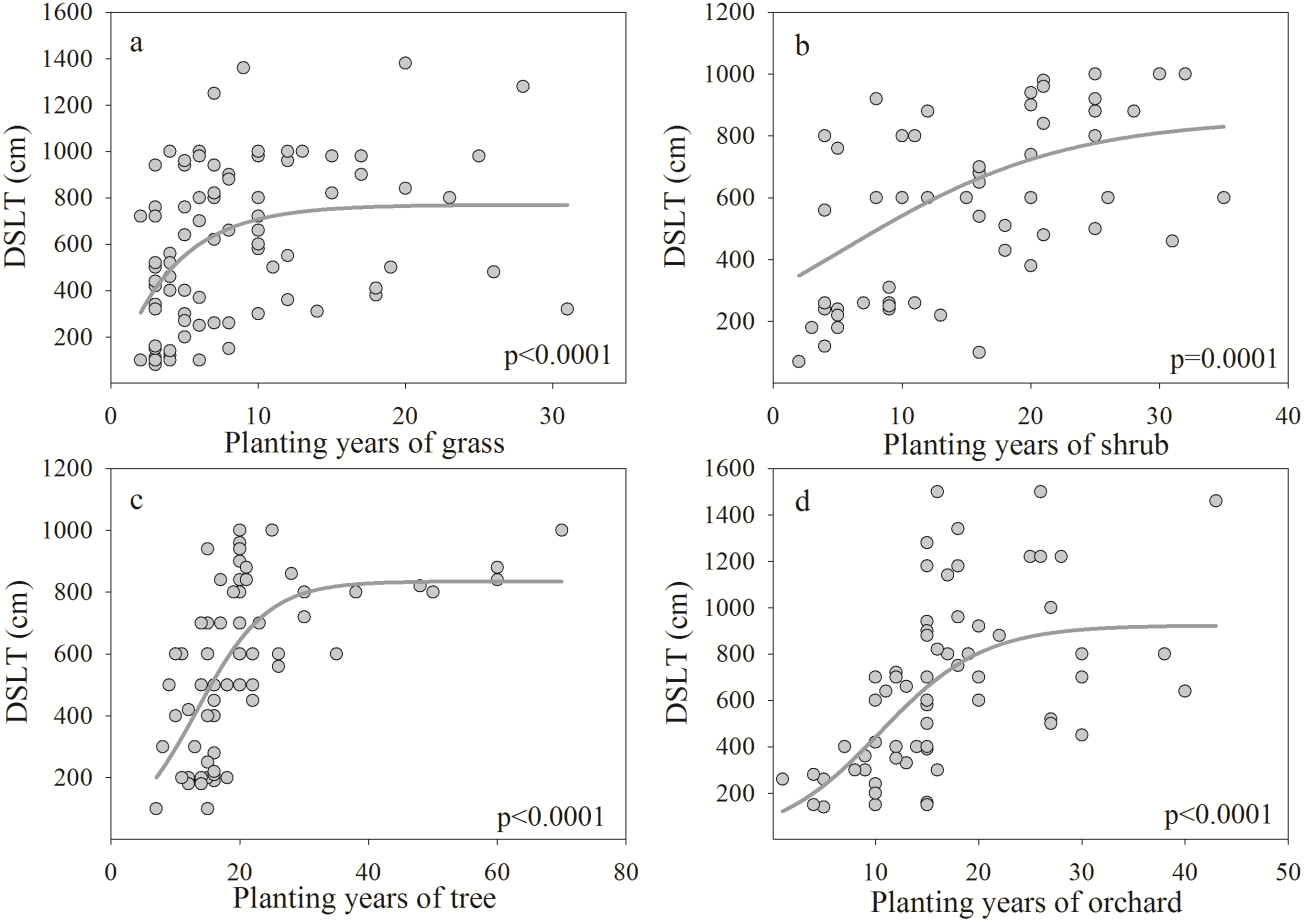
Relationship between the dry soil layer thickness (DSLT) and planting years for different vegetation types.

**Fig 6:**
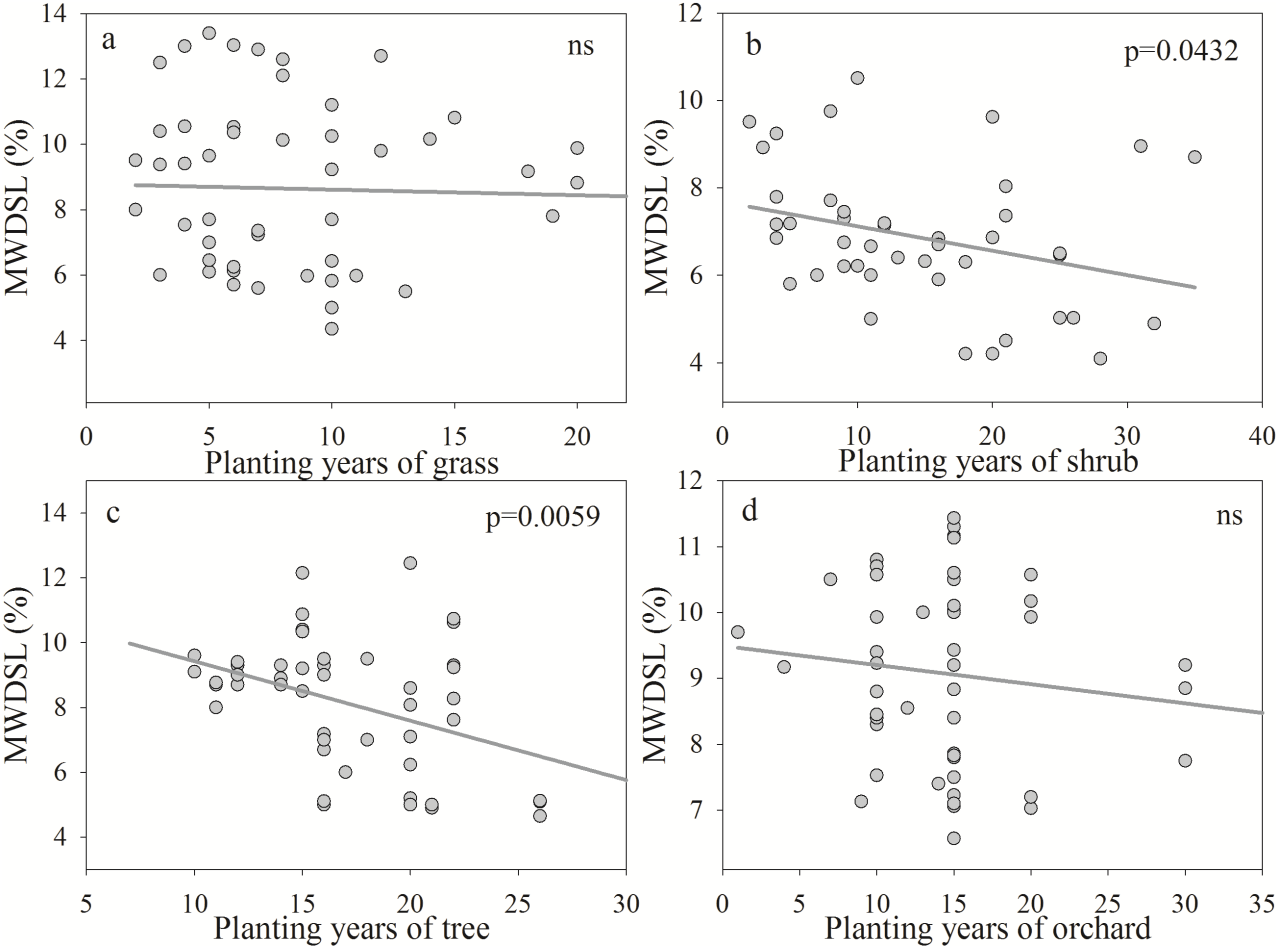
Relationship between the mean water content of the dry soil layer (MWDSL) and planting years for different vegetation types.

The DSLFD presented a significant negative correlation with the number of years after planting and decreased with increasing years (Fig 4). The planting years explained 11.34%, 12.19%, 19.08% and 11.13% of the variability in the DSLFD in grasses, shrubs, trees and orchards, respectively. The DSLT showed a positive correlation with planting years and a steeper increase during the period of rapid growth (Fig 5). The sharp increase occurred until approximately 8, 18, 22 and 18 years for grasses, shrubs, trees and orchards in the Loess Plateau, respectively. Planting years were able to explain 16.68%, 26.41%, 39.07% and 31.29% of the variability in the DSLT in grasses, shrubs, trees and orchards, respectively. The MWDSL decreased with increasing planting years (Fig 6) and exhibited a significant negative correlation under shrubs and trees. Planting years were able to explain 9.38% and 14.47% of the variability in the MWDSL in shrubs and trees, respectively. These results indicate that planting years contributed to the development of DSLs, which is consistent with the results of previous studies [8, 10, 39].

Alfalfa has been planted on a large scale as a perennial pasture on the Loess Plateau. As a high-consumption plant, the rate of elongation of its roots can reach 1.12 m per year [43]. Under alfalfa, a DSL developed within three years, the DSLT increased, the MWDSL decreased with growth [43], and the DSL exceeded 20 m after 23 years [32]. In addition, apple trees have been planted on a large scale on the Loess Plateau for economic profit, and these trees now account for more than half of the apple trees in China and have become a focus in recent years [6–8, 12, 39, 44]. Wang et al. [39] reported that the DSLFD of a 5-year-old apple orchard was approximately 80 cm, whereas the DSLFDs of 12- and 18-year-old orchards were only 30 and 20 cm, respectively. Additionally, the DSLT increased with increasing apple orchard age in the order 5 years (120 cm) < 12 years (620 cm) < 18 years (>900 cm). Based on water balance equations and the characteristics of the water cycle, Huang, Yang and Li [45] reported that 8-, 15- and 28-year-old apple trees in Luochuan County excessively used stored water in the 0–1,000 cm soil layer by as much as 151.0 mm, 762.9 mm and 785.6 mm, respectively.

With growth, plants develop larger root systems and consume more water to obtain more net primary production. Li and Huang [46] and Wang et al. [12] reported that the formation and development of a DSL in scrubland or grassland were significantly correlated with planting years. Thus, accounting for planting years is necessary when studying the development or recovery of DSLs, soil water management and water balance on the Loess Plateau or other water-limited regions around the world [39].

### SWC under different planting years of apple and alfalfa

**Fig 7:**
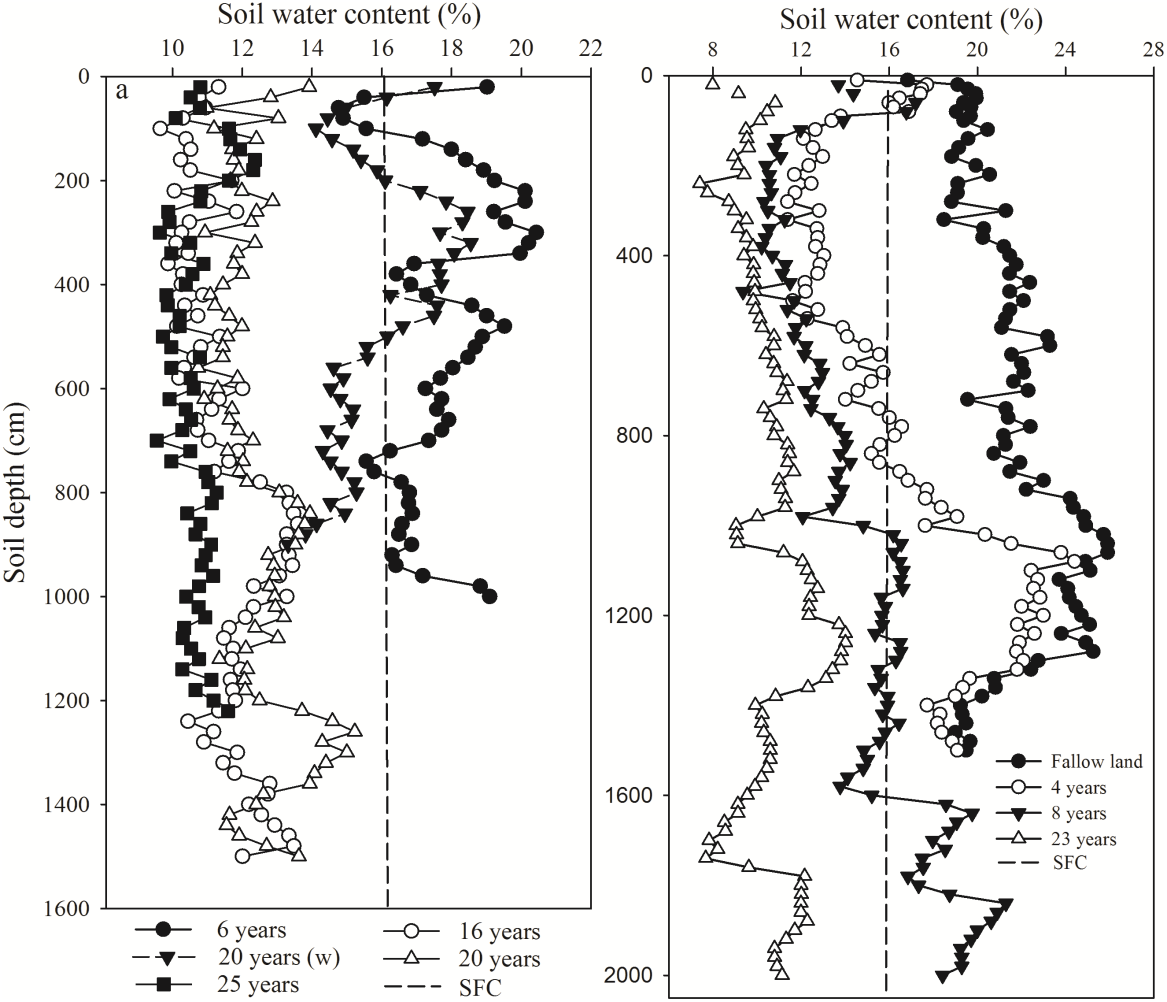
The SWC after various years since planting: (a) apple orchard; and (b) alfalfa. Legends were planting years and (w) represent with irrigation. The dotted line represents the stable field capacity (SFC). The figures’ data are based on Cao et al. [52] (a), Cheng and Liu [32] and Cheng et al. [53] (b).

The profile distribution of SWC under apple and alfalfa with different planting years (Fig 7) shows that the SWC under both apple and alfalfa decreased gradually with increasing planting years and corresponded with changes in depth, which implies that the DSL in the profile may be a function of planting years. As the plant community develops, the soil water demands of the plants increase.

Previous studies have reported that the SFC (dotted line in Fig 7) is the upper SWC for a DSL [7, 8, 10, 12, 47], which facilitates determining whether a soil layer belongs to a DSL. In Fig 7, the SWC was sufficiently low to form a DSL under both apple and alfalfa, although the formation and development of DSLs under the two vegetation types differed. Under apple trees, a weak DSL was formed by the sixth year following planting, whereas under alfalfa, a strong DSL was formed in the fourth year. These differences can be attributed to differences in the biological characteristics of the two vegetation types because the vigorous growth period of alfalfa, which occurs earlier than that of apples and features stronger evapotranspiration. The DSLT exceeded 1,500 cm after 16 years of apple tree growth (Fig 7a), whereas it reached 1,000 cm after 8 years of alfalfa growth and exceeded 2,000 cm after 23 years; however, a DSL did not occur in the soil profile of fallow land (Fig 7b). Different vegetation types had different impacts on the hydrological cycle through water uptake via plant roots, evapotranspiration and the canopy layer effect [12, 37], and these factors varied during different growth period of the same vegetation type [12, 48]. Consequently, the selection of an appropriate plant type is important for maintaining the “soil water pool” [6, 13, 19] during the process of vegetation restoration on the Loess Plateau. Thus, to prevent or reduce DSL formation in orchards and alfalfa plantations, supplemental irrigation and biomass reductions according to the water carrying capacity of the soil may be an effective management measure for maintaining high productivity and sustainable development in this area.

### Relationship between the SWC, DSLT and MWDSL and wheat yield

**Fig 8:**
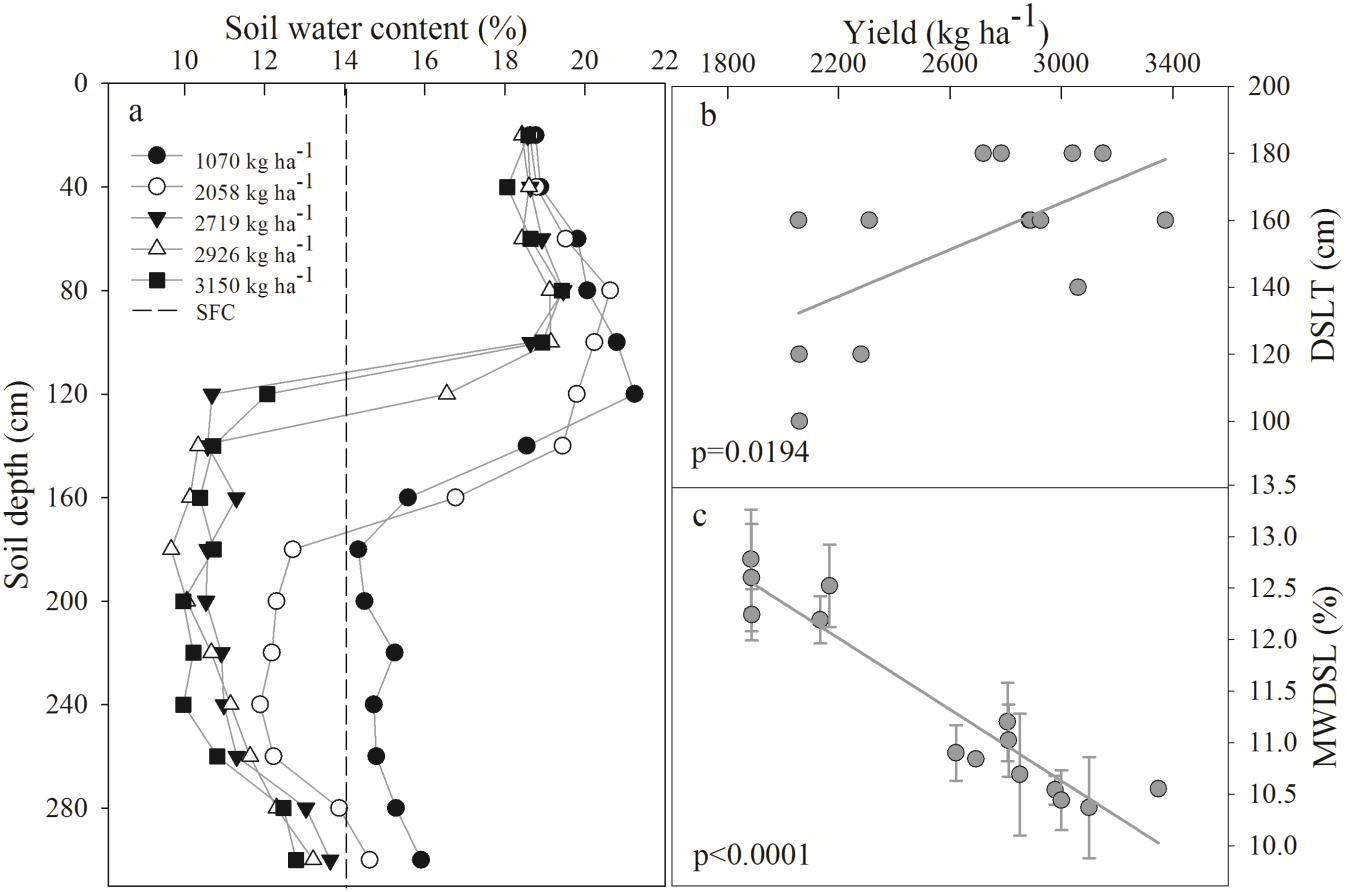
Dry soil layer under different yields of wheat: (a) SWC under different yields; (b) relationship between the dry soil layer thickness (DSLT) and yield; (c) relationship between the mean water content of the dry soil layer (MWDSL) and yield. The dotted line represents the stable field capacity (SFC). The data are based on Xue and Hao [54].

The SWC decreased with increasing wheat yields and declined significantly in the 0–120 cm layer because the main water-consuming layer of wheat was distributed at 120 cm in this area [49]. DSLs were not observed in low-yield areas (1,070 kg ha^-1^), but with the continuous increasing of yield, the SWC was lower than the SFC (Fig 8a). Significant correlations were observed between the yield and the DSLT (p = 0.0194) and MWDSL (p<0.0001), with wheat yield explaining 37.76% of the DSLT and 90.20% of the MWDSL (Fig 8b and 8c). The development of DSLs was stronger with increasing yield, which is consistent with the results of Li et al. [50], who reported that the DSLT in a high-yield wheat field reached an average of 560 cm (ranging from 40 to 600 cm), whereas the DSLT in a low-yield field only averaged 220 cm (ranging from 80 to 300 cm), and the MWDSL values of the high- and low-yield fields were 12.63% and 12.96%, respectively. Wheat appears to absorb deep soil water to obtain a high yield in this region because precipitation cannot meet the requirements for wheat crops. However, the occurrence of DSLs in farmland introduces a challenge for food security because of deficits in the “soil water-pool,” which increases the dependence of crops on precipitation.

### Formation of DSLs and influencing factors

The formation of a DSL is a comprehensive symptom of plant-soil-atmosphere interactions that lead to a negative water balance. DSLs are influenced by local climate (precipitation) [6, 8, 14, 17], topographical factors (slope aspect, slope position, etc.) [6, 7, 17], soil properties (soil texture, soil water holding ability, etc.) [10, 14, 39], land use [23, 37, 44], and plant characteristics (vegetation coverage, planting years, exorbitant productivity and density) [6, 12, 37, 46]. The present study evaluated the effects of a large-scale factor (precipitation) on DSL development as well as the effects of large- and local-scale factors (vegetation type, planting years and yield) on the patterns of DSL distribution. These factors were found to significantly affect the development of DSLs. By understanding the impact of precipitation, vegetation type, planting years and yield on the formation of DSLs on the Loess Plateau, certain effective measures can be applied to mitigate this process and maintain sustainable development of this region when implementing soil erosion controls and re-vegetation projects. Native species should be promoted for planting in this region because they present a significantly lower degree of DSL formation than non-native species [51]. In addition, certain land-use management factors, such as decreasing plant density, reducing planting years, increasing plant diversity, cutting grass and rotating crops, should be fully considered because they may help maintain soil water storage for plants under normal growth conditions during periods of drought, particularly under the threat of increasing climate change.

## Conclusions

The present study investigated the development of DSLs (represented by the DSLT, DSLFD, and MWDSL) across the Loess Plateau. The development of DSLs differed greatly according to the levels of precipitation and types of vegetation on the Loess Plateau. Higher mean annual precipitation leads to higher DSLFD and MWDSL and lower DSLT. Crops presented lower DSLT and higher MWDSL, whereas significant differences were not observed in the DSLT among grasses, trees, shrubs and orchards. Furthermore, the extent of the DSL development was significantly correlated with the planting years and wheat yields. Understanding the relationship between DSLs and climatic and plant factors on the Loess Plateau is useful for land management and provides guidance for DSLs mitigation or recovery through changes in the land-use type and utilization of more appropriate plant species selections and management practices. The results of this study will provide a reference for other similar regions around the world. Furthermore, the results of the present study may be helpful for understanding the eco-hydrological processes in water-limited ecosystems.

## Supporting Information

S1 File Database used in the study. (XLS)

S1 Table PRISMA (Preferred Reporting Items for Systematic Reviews and Meta-Analyses) checklist. (DOC)

## References

[1] Giorgi F, Bi X. Regional changes in surface climate interannual variability for the 21st century from ensembles of global model simulations. Geophys Res Lett. 2005; 32: 13701.1–5.

[2] Breshears DD, Cobb NS, Rich PM, Price KP, Allen CD, Balice RG, et al Regional vegetation die-off in response to global-change-type drought. Proc Natl Acad Sci USA. 2005; 102: 15144–15148. 16217022 10.1073/pnas.0505734102 PMC1250231

[3] Brown K. Water scarcity: forecasting the future with spotty data. Science. 2002; 297: 926–927. 12169710 10.1126/science.297.5583.926

[4] Easterling DR, Meehl GA, Parmesan C, Changnon SA, Karl TR, Mearns LO. Climate extremes: observations, modeling, and impacts. Science. 2000; 289: 2068–2074. 11000103 10.1126/science.289.5487.2068

[5] Zavaleta ES, Thomas BD, Chiariello NR, Asner GP, Shaw MR, Field CB. Plants reverse warming effect on ecosystem water balance. Proc Natl Acad Sci USA. 2003; 100: 9892–9893. 12907704 10.1073/pnas.1732012100 PMC187878

[6] Shangguan Z. Soil desiccation occurrence and its impact on forest vegetation in the Loess Plateau of China. Int J Sust Dev World. 2007; 14: 299–306.

[7] Wang L, Wang Q, Wei S, Shao M, Li Y. Soil desiccation for Loess soils on natural and regrown areas. For Ecol Manage. 2008; 255: 2467–2477.

[8] Wang Y, Shao M, Liu Z. Large-scale spatial variability of dried soil layers and related factors across the entire Loess Plateau of China. Geoderma. 2010; 159: 99–108.

[9] Yang W, Shao M, Peng X, Xia W. On the relationship between environmental aridization of the Loess Plateau and soil water in loess. Sci China Ser D. 1999; 42: 240–249.

[10] Li YS. The properties of water cycle in soil and their effect on water cycle for land in the loess region. Acta Ecol Sin. 1983; 3: 91–101.

[11] Jipp PH, Nepstad DC, Cassel D, De Carvalho CR. Deep soil moisture storage and transpiration in forests and pastures of seasonally-dry Amazonia Potential Impacts of Climate Change on Tropical Forest Ecosystems. Springer 1998; pp. 255–272.

[12] Wang Y, Shao M, Shao H. A preliminary investigation of the dynamic characteristics of dried soil layers on the Loess Plateau of China. J Hydrol. 2010; 381: 9–17.

[13] Yang WZ. Soil water resources and afforestation in Loess Plateau. J Nat Resour. 2001; 16: 433–438.

[14] Wang Y, Shao M, Liu Z, Warrington DN. Investigation of factors controlling the regional-scale distribution of dried soil layers under forestland on the Loess Plateau, China. Surv Geophys. 2012; 33: 311–330.

[15] Robinson N, Harper R, Smettem K. Soil water depletion by Eucalyptus spp. integrated into dryland agricultural systems. Plant Soil. 2006; 286: 141–151.

[16] Richards B, Peter P, Emerson W. The effects of vegetation on the swelling and shrinking of soils in Australia. Geotechnique. 1983; 33: 127–139.

[17] Chen H, Shao M, Li Y. Soil desiccation in the Loess Plateau of China. Geoderma. 2008; 143: 91–100.

[18] Chen H, Shao M, Li Y. The characteristics of soil water cycle and water balance on steep grassland under natural and simulated rainfall conditions in the Loess Plateau of China. J Hydrol. 2008; 360: 242–251.

[19] Li YS. Effects of forest on water circle on the Loess Plateau. J Nat Resour. 2000; 16: 427–432.

[20] Shangguan Z, Zheng S. Ecological properties of soil water and effects on forest vegetation in the Loess Plateau. Int J Sust Dev World. 2006; 13: 307–314.

[21] Fu M, Qian W, Niu, Ma G. Impact of the continuous drought on the depth of dry soil layer and on the existence of plants. Arid Zone Research. 2001; 19: 71–74.

[22] Yang W. The preliminary discussion on soil desiccation of artificial vegetation in the northern regions of China. Sci Silvae Sin. 1996; 32: 78–85.

[23] Han RL, Hou QC. An Analysis of Genesis of Small Aged Trees on the Loess Plateau. Agric Res Arid Areas. 1996; 14: 104–108.

[24] Wang G, Innes JL, Lei J, Dai S, Wu SW. China's forestry reforms. Science. 2007; 318: 1556–1557. 18063773 10.1126/science.1147247

[25] Han SF, Li YS, Shi YJ, Yang XM, Zhang XZ, Shi ZY. The Characterstics of Soil Moisture Resources on the Loess Plateau. Bull Soil Water Conserv. 1990; 10: 36–43.

[26] Wang L, Shao MA, Hou QC. Preliminary research on measured indexes of dried soil layer. J Soil Water Conserv. 2000; 14: 87–90.

[27] Chen BQ, Zhao JB, Li YH. Research on causes of dried soil layer in the Loess Plateau. Geography Geo-Information Sci. 2009; 25: 85–91.

[28] Zhang H, Wang YP, Gao PC, Sun PY. Research on mechanism about forming dry layer of slope land and way of supplying water on Loess plateau. J Soil Water Conserv. 2003; 17: 162–164.

[29] Fu B, Wang J, Chen L, Qiu Y. The effects of land use on soil moisture variation in the Danangou catchment of the Loess Plateau, China. Catena. 2003; 54: 197–213.

[30] Liu P, Hao W, Li J, Jia Z. Study on curve fitting features of soil moisture and root system's dynamic distribution in alfalfa grassland in drought areas of southern Ningxia. J Agr University Hebei. 2011; 34: 29–34.

[31] Cheng JM, Wan HE, Wang J, Yong SP. Growth of Caragana korshinskii and Depletion Process of Soil Water in Semi-Arid Region. Sci Silvae Sin. 2005; 41: 37–42.

[32] Cheng L, Liu W. Soil moisture distribution in deep layers and its response to different land use patterns on Loess Tableland. Trans CSAE. 2011; 27: 203–207.

[33] Huang M, Gallichand J. Use of the SHAW model to assess soil water recovery after apple trees in the gully region of the Loess Plateau, China. Agric Water Manage. 2006; 85: 67–76.

[34] Zhu X, Li Y, Peng X, Zhang S. Soils of the loess region in China. Geoderma. 1983; 29: 237–255.

[35] Shi H, Shao M. Soil and water loss from the Loess Plateau in China. J Arid Environ. 2000; 45: 9–20.

[36] Chen L, Wei W, Fu B, Lü Y. Soil and water conservation on the Loess Plateau in China: review and perspective. Prog Phys Geog. 2007; 31: 389–403.

[37] Wang Z, Liu B, Zhang Y. Soil moisture of different vegetation types on the Loess Plateau. J Geogr Sci. 2009; 19: 707–718.

[38] Wang L, Shao M, Wang Q, Jia Z, Li J. Review of research on soil desiccation in the Loess Plateau. Trans CSAE. 2004; 20: 27–31.

[39] Wang Y, Shao M, Zhu Y, Liu Z. Impacts of land use and plant characteristics on dried soil layers in different climatic regions on the Loess Plateau of China. Agr Forest Meteorol. 2011; 151: 437–448.

[40] Kattge J, Knorr W. Temperature acclimation in a biochemical model of photosynthesis: a reanalysis of data from 36 species. Plant Cell Environ. 2007; 30: 1176–1190. 17661754 10.1111/j.1365-3040.2007.01690.x

[41] Yao YB, Wang YR, Li YH, Zhang XY. Climate warming and drying and its environmental effects in the Loess Plateau. Resour sci. 2005; 27: 146–152.

[42] Wang L, Shao M, Zhang Q. Distribution and characters of soil dry layer in north Shaanxi Loess Plateau. Chinese J Appl Ecol. 2004; 15: 436–442. 15233111

[43] Cheng J, Wan H, Wang J. Alfalfa growth and its relation with soil water status in loess hilly and gully region. Chinese J Appl Ecol. 2005; 16: 435–438. 15943352

[44] He FH, Jiang WG, Huang MB. Ecological water effect of returning orchard to cultivated land in apple base of gully region of the Loess Plateau. Geographical Res. 2010; 29: 1863–1869.

[45] Huang M, Yang X, Li Y. Effect of apple base on regional water cycle in Weibei upland of the Loess Plateau. Acta Geographica Sinica. 2001; 56: 12–22.

[46] Li Y, Huang M. Pasture yield and soil water depletion of continuous growing alfalfa in the Loess Plateau of China. Agr Ecosyst Environ. 2008; 124: 24–32.

[47] Wang X, Sun G, Jia Y, Li F, Xu J. Crop yield and soil water restoration on 9-year-old alfalfa pasture in the semiarid Loess Plateau of China. Agric Water Manage. 2008; 95: 190–198.

[48] Cheng JM, Wan HE. Vegetation construction and soil and water conservation in the Loess Plateau of China. China Forestry Publishing House, Beijing (in Chinese). 2002; pp. 101–125.

[49] Zhong Y, Shangguan Z. Water Consumption Characteristics and Water Use Efficiency of Winter Wheat under Long-Term Nitrogen Fertilization Regimes in Northwest China. PloS one. 2014; 9: e98850 doi: 10.1371/journal.pone.0098850 24905909 10.1371/journal.pone.0098850 PMC4048222

[50] Li J, Jiang B, Hu W, Ciren YJ, Zhao YJ, Li XF, et al Characteristics of deep soil desiccation on rainfed grain croplands in different rainfall areas of the Loess Plateau of China. J Nat Resour. 2009; 24: 2124–2134.

[51] Wang ZQ, Liu BY, Hai CX, Fu JS. Analysis of soil water content of different vegetation types in the North-Western part of Shanxi Province. J Arid Land Resour Environ. 2002; 16: 53–59.

[52] Cao Y, Li J, Zhang S, Wang Y, Cheng K, Wang X, et al Characteristics of deep soil desiccation of apple orchards in different weather and landform zones of Loess Plateau in China. Trans CSAE. 2012; 28: 72–79.

[53] Cheng L, Liu W, Li Z. Soil water in deep layers under different land use patterns on the Loess Tableland. Acta Ecologica Sinica. 2014; 8: 1975–1983.

[54] Xue X, Hao M. Study on the relationship between fertilization, yield and soil desiccation in the Loess Plateau region. Agril Res Arid Areas. 2010; 6: 75–81.

